# CAD v1.0: Cancer Antigens Database Platform for Cancer Antigen Algorithm Development and Information Exploration

**DOI:** 10.3389/fbioe.2022.819583

**Published:** 2022-05-12

**Authors:** Jijun Yu, Luoxuan Wang, Xiangya Kong, Yang Cao, Mengmeng Zhang, Zhaolin Sun, Yang Liu, Jing Wang, Beifen Shen, Xiaochen Bo, Jiannan Feng

**Affiliations:** ^1^ State Key Laboratory of Toxicology and Medical Countermeasures, Beijing Institute of Pharmacology and Toxicology, Beijing, China; ^2^ Beijing Key Laboratory of Therapeutic Gene Engineering Antibody, Beijing, China; ^3^ State Key Laboratory of Toxicology and Medical Countermeasures, Beijing Key Laboratory of Neuropsychopharmacology, Beijing Institute of Pharmacology and Toxicology, Beijing, China; ^4^ Beijing Geneworks Technology Co., Ltd., Beijing, China; ^5^ Center of Growth, Metabolism and Aging, Key Laboratory of Bio-Resource and Eco-Environment of Ministry of Education, College of Life Sciences, Sichuan University, Chengdu, China; ^6^ Beijing Capital Agribusiness Future Biotechnology Co, Beijing, China; ^7^ Department of Biotechnology, Beijing Institute of Radiation Medicine, Beijing, China

**Keywords:** tumor-associated antigens (TAAs), tumor-specific antigens (TSAs), prediction model, cancer antigen, neoantigen

## Abstract

Cancer vaccines have gradually attracted attention for their tremendous preclinical and clinical performance. With the development of next-generation sequencing technologies and related algorithms, pipelines based on sequencing and machine learning methods have become mainstream in cancer antigen prediction; of particular focus are neoantigens, mutation peptides that only exist in tumor cells that lack central tolerance and have fewer side effects. The rapid prediction and filtering of neoantigen peptides are crucial to the development of neoantigen-based cancer vaccines. However, due to the lack of verified neoantigen datasets and insufficient research on the properties of neoantigens, neoantigen prediction algorithms still need to be improved. Here, we recruited verified cancer antigen peptides and collected as much relevant peptide information as possible. Then, we discussed the role of each dataset for algorithm improvement in cancer antigen research, especially neoantigen prediction. A platform, Cancer Antigens Database (CAD, http://cad.bio-it.cn/), was designed to facilitate users to perform a complete exploration of cancer antigens online.

## Introduction

Colev (189) was the first to attempt to leverage patient immune systems to fight against cancer. Since then, immunotherapy has made great progress, especially in immune checkpoint blockades of cytotoxic T-lymphocyte-associated protein 4 and programmed cell death protein 1 (PD-1) in melanoma (Hodi et al., 2010; Robert et al., 2015) and other cancers (Zou et al., 2016). However, not all patients benefit from immune checkpoint inhibitors, which may cause immune-related adverse events. Therefore, additional immunotherapies need to be developed.

Recently, cancer vaccines have attracted increasing attention with promising results in preclinical studies (Castle et al., 2012; Gubin et al., 2014) and clinical outcomes (Li et al., 2017; Ott et al., 2017; Sahin et al., 2017) in individual or combination therapies. Cancer antigens are peptides in tumor cells present in antigen-presenting cells, which then provoke T-cell activation to kill tumor cells. The process of immune tumor killing is displayed in Supplementary Figure S1A. Ehx and Perreault (2019) suggested classifying cancer antigens into the following three categories based on specificity and mutation situations: tumor-associated antigens (TAAs), aberrantly expressed tumor-specific antigens (aeTSAs), and mutated tumor-specific antigens (mTSAs). TAAs are typically proteins that are overexpressed in tumor cells but also exist in normal tissues. aeTSAs drive the majority of epigenetic changes in atypical translation events, which are widely expressed in various cancers and could be shared among patients (Probst et al., 2017; Schuster et al., 2017; Gee et al., 2018; Laumont et al., 2018). mTSAs, also known as neoantigens, are patient-specific mutation peptides that only exist in tumor cells. The classification method was also used to organize recruited cancer antigens in the current study.

The efficacies of many TAAs and aeTSAs have been investigated in clinical trials to date, but global clinical outcomes have not been encouraging. However, many neoantigen-based cancer vaccines have generated effective antitumor responses in multiple preclinical studies (Gubin et al., 2014; Li et al., 2017). For example, Castle et al. (2012) identified 16 candidate neoantigens confirmed to be immunogenic using IFN-gamma enzyme-linked immunospot assay. Peptide immunization has conferred *in vivo* tumor control in protective and therapeutic settings. Apart from allograft transplantation models, the establishment of immunodeficient nude and NOD.scid.gamma mice make it possible to study human cancer cells in mice. Zhang et al. (2017) found that two neoantigens, ROBO3 A1265V and PALB2 H198D, with adoptive transfer of autologous peripheral blood mononuclear cells stimulated *in vitro* with mutant peptides decreased tumor growth. In addition, many recent preclinical studies have achieved a broad immune response, such as increased cytotoxic T-lymphocyte response and decrease in the tumor growth rate in mouse models (Ahn et al., 2015; Chen et al., 2015; Rekoske et al., 2015; Lambricht et al., 2016; Lopes et al., 2017, 2018; Wu et al., 2017; Zhao et al., 2017; Gao et al., 2018).

In addition to the preclinical research mentioned earlier, cancer neoantigen vaccines have also made great progress in clinical research. Numerous clinical studies have confirmed the safety and efficacy of cancer neoantigen vaccines (Ott et al., 2017, 2020; Keskin et al., 2019; Platten et al., 2021), and the outcomes of clinical trials on melanoma were particularly encouraging. Ott et al. (2017) demonstrated the potential of neoantigen vaccines in the treatment of melanoma. Of six vaccinated patients after surgical resection, four had no recurrence at the 20- to 32-month follow-up; the remaining two with recurrence were treated with PD-1. After antibody therapy, complete remission was achieved. Keskin et al. (2019) (NC02287428) found that neoantigen-specific T cells from peripheral blood can migrate into intracranial glioblastoma, which provided evidence that cancer neoantigen vaccines could enhance the immune microenvironment of glioblastoma cells. These clinical studies have indicated that neoantigen vaccines could play a meaningful role in different tumors. This evidence provides great confidence for further exploration of the clinical path of neoantigen vaccines.

In both preclinical and clinical studies, rapid and accurate neoantigen prediction plays a key role in the development of neoantigen vaccines. In this study, we explore the use of relevant datasets in neoantigen prediction.

The process of neoantigen prediction includes 1) sampling of patient tumor and adjacent tissues, 2) whole-genome sequencing or whole-exome sequencing and RNA-seq sequencing, 3) identifying somatic mutations and calculating the expression levels and HLA subtypes, 4) calculating the binding affinity of potential neoantigens to HLA subtypes, and 5) screening candidate neoantigen. A flowchart of neoantigen prediction is displayed in Supplementary Figure S1B.

Currently, multiple software programs and algorithms have been developed in neoantigen prediction pipelines (Soria-Guerra et al., 2015; Peng et al., 2019; Lancaster et al., 2020), and the most commonly used tools are NetMHC (Lundegaard et al., 2008a) and NetMHCpan (Reynisson et al., 2021), which use allele-specific epitope prediction and pan-specific machine learning methods, respectively. After affinity screening, researchers further screen candidate neoantigens by peptide immunogenicity prediction, such as neoantigen prediction workflows INeo-Epp (Wang et al., 2020) and pTuneos (Chi Zhou et al., 2019). These algorithms are based on the immunogenicity-related characteristics of T-cell epitope peptides combined with machine learning algorithms.

Insufficient and low-quality datasets affect the accuracy of prediction models. Presently, many cancer antigens or neoantigen peptides have been generated. It is necessary to recruit and organize these datasets to facilitate the improvement of existing algorithms or the development of new algorithms. Existing databases help accomplish this purpose. The Cancer–Testis Database (Almeida et al., 2009) is a knowledge base of high-throughput and curated data on cancer–testis antigen a. It provides an important resource for the exploration of cancer–testis antigens. NEPdb (Xia et al., 2021), dbPepNeo (Tan et al., 2020), and NeoPeptide (Wei-Jun Zhou et al., 2019) contain curated neoantigens but lack any other types of cancer antigens; TSNAdb v1.0 (Wu et al., 2018) predicts candidate neoantigens based on The Cancer Genome Atlas and The Cancer Immunome Atlas datasets, which are useful for comparing candidate neoantigens in different cancers. The Tumor T-cell Antigen Database, TANTIGEN 2.0 (Zhang et al., 2021), contains HLA ligands and T-cell epitopes and classifies cancer antigens. However, this database does not contain invalid peptides and lacks clinically relevant data. The blueprint of the Cancer Epitope Database and Analysis Resource (CEDAR) database, proposed by Koşaloğlu-Yalçın et al. (2021), is based on the Immune Epitope Database (IEDB) and tries to collect more specific information and classify it in detail. The aforementioned databases focus on online information exploration but not on algorithm development. To fill this gap, we built the Cancer Antigens Database (CAD), which recruited all cancer antigen peptides and relevant datasets, established neoantigen simulation datasets, carried out detailed data preprocessing, and explained the scope of application and precautions of different datasets in algorithm development. A user-friendly platform was concurrently established to facilitate online exploration.

## Data Collection

We recruited cancer antigens verified from published articles (∼900) and collected associated peptides from other resources, such as the IEDB (Vita et al., 2019) for peptide binding and T-cell epitope datasets, the SysteMHC atlas (Shao et al., 2018) for mass spectrometry (MS) datasets, the VDJdb database (Bagaev et al., 2020) for antigens with T-cell receptor (TCR) sequence datasets, and the Protein Data Bank for three-dimensional structure of peptide-major histocompatibility complex (p-MHC) or pMHC–TCR complex. After recruiting all verified cancer antigens, a small amount of neoantigen data were insufficient for the development of algorithms; therefore, we also generated simulation neopeptides, for which dbSNP datasets from the National Center for Biotechnology Information (Sherry et al., 2001), the reviewed SwissProt human proteins sequence from Uniprot (Bateman et al., 2021), and verified T-cell epitopes from IEDB were used.

Finally, more than 800 cancer antigens were recruited for our database, which includes cancers such as skin, brain, and kidney cancers. as well as a total of 267 verified neoantigens. Except for verified cancer antigens, information on associated peptides, including cleaned MHC–peptide binding (569953), T-cell epitopes (66151), pMHC MS (509536), antigens with TCR sequences (60267), and more than 6,000 simulated neopeptides, was included in our datasets (only HLA-A*0201 was included; for more HLA alleles, users can generate their own HLA alleles datasets. Refer to methods described in Supplementary Figure S2 and code from https://github.com/yujijun/NeoSimData).

## Usage About Datasets

The majority of datasets in our database are suitable for algorithm development. For example, all curated verified cancer antigen datasets can be used as preliminary verification for predicting candidate antigens to test whether the same or similar cancer antigens have been studied or reported. The simulated neopeptide datasets have been used in feature-based neoantigen immunogenicity algorithms (Kim et al., 2018). A large number of neoantigen simulation datasets have greatly filled the gap of insufficient neoantigen datasets, providing a choice for the application of machine learning and even deep learning in the immunogenicity prediction of neoantigens. We provided an upgraded version to facilitate the generation of more flexible and widely applicable simulation datasets.

Part of the MHC-binding datasets in IEDB has been used in binding prediction algorithms; it should be noted that many peptides from IEDB belonged to bacteria or viruses but not humans and also were not obtained by standardized experimental methodologies in cancer models, which may reduce the accuracy of the algorithm prediction to a certain extent (Jiang et al., 2019). In addition, redundant information can also cause inaccurate model evaluation. Therefore, cleaned and selected human origin datasets have been generated and stored in our database. At present, tumor neoantigen prediction and filtering are mainly based on the binding affinity of MHC and peptides. However, peptide and MHC binding is a necessary but not sufficient condition for T-cell activation; therefore, many peptides screened by peptide–MHC binding cannot activate T cells to induce immune responses. T-cell epitope datasets were a useful resource to predict peptide immunogenicity for cancer treatment. For example, T-cell epitope datasets could be used as prior knowledge to improve the accuracy of algorithms, just as Balachandran et al. (2017) and Luksza et al. (2017) developed a new approach for assessing whether a tumor is immunogenically based on estimated likelihood of TCR recognition for each predicted neoantigen. These estimates were computed from sequence similarities between the predicted neoantigens and a dataset of immunogenic epitopes. Wang et al. (2020) developed INeo-Epp, a random forest classifier for T-cell immunogenic HLA-I-presenting antigen epitopes and neoantigens based on sequence-related amino acid features. Riley et al. (2019) trained a neural network on structural features that influence TCR and peptide-binding energies.

Several groups are interested in systematically studying the binding of TCR to peptides/MHC. Gielis et al. (2020) developed a web tool TCRex for the prediction of T-cell receptor sequence epitope specificity, which allows users to upload TCR sequences and predict interaction with multiple known epitopes; Montemurro et al. (2021) developed NetTCR-2.0, which enables accurate prediction of TCR–peptide binding by the “shallow” convolutional neural network; Jokinen et al. (2021) developed TCRGP, a novel Gaussian process method that predicts recognition between T-cell receptors and epitopes, which has better performance in algorithm evaluation than existing state-of-the-art methods in epitope specificity predictions. Some databases have been built for curating such research; for example, the VDJdb database (Bagaev et al., 2020) curates TCR sequences with known antigen specificities. Peptide information of the dataset has also been integrated into our website.

Finally, we compiled a list of benchmark datasets in our database, which could be used for testing and verification of neoantigen pipelines. It included the entire process from original sequence datasets, predicted neoantigens, and experimentally verified immunogenic peptides, which have been used for the evaluation of several complete neoantigen prediction platforms such as pVACtools (Hundal et al., 2020), pTuneo (Chi Zhou et al., 2019), and neoepiscope (Wood et al., 2020). All these datasets can also be used for cross-sectional evaluation and comparison between different neoantigen prediction pipelines (Supplementary Table S1). Detailed datasets statistics and usage are shown in Supplementary Table S2, and all the datasets mentioned before can be downloaded from our database (http://cad.bio-it.cn/#/Download).

## Construction of Cancer Antigen Platform

**FIGURE 1:**
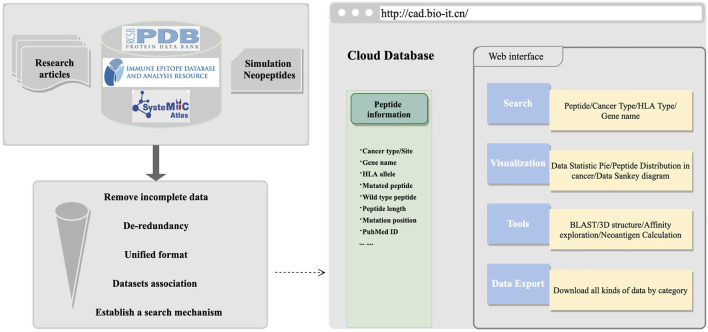
Schematic diagram of data preprocessing and website architecture.

These datasets can be used in algorithm development and exploration of cancer antigens online. After all datasets were collected or generated, we organized the information into a unified format, including tumor name, tissue site, gene name, peptides, MHC alleles, and mutation information, and then, a user-friendly retrieval mechanism was established. The HOME page had detailed statistics about peptide information. On the SEARCH page, multiple retrieval methods were established. To facilitate users to perform comprehensive peptide exploration, we provided information such as hydrophobic and hydrophilic properties, which have been proved to have an important influence on the immunogenicity of antigens (Chowell et al., 2015). We also integrated the sequence alignment program of BLAST (Zeng et al., 2007) for sequence similarity exploration and constructed pMHC–TCR protein structure modeling tools for structure interaction exploration. To conduct a more in-depth analysis of cancer antigens, especially neoantigens, we introduced and linked some of the commonly used epitopes prediction (Buus et al., 2003; Nielsen et al., 2003, 2007a; Peters et al., 2003; Peters and Sette, 2005; Tenzer et al., 2005; Lundegaard et al., 2006, 2008b, 2008a), affinity prediction (Sturniolo et al., 1999; Nielsen et al., 2007b; Sidney et al., 2008; Wang et al., 2008, 2010; Nielsen and Lund, 2009; Andreatta et al., 2015; Jensen et al., 2018; Reynisson et al., 2021), and neoantigen prediction pipelines in the TOOLS page; all database or software mentioned is organized in Supplementary Table S3. The schematic diagram framework of the web construction processes is shown in Figure 1. Cancer antigen researchers are encouraged to use this platform to submit relevant information about cancer antigens on the SUBMIT page and feel free to download interesting datasets on the DOWNLOAD page. More information about this website can be found at http://cad.bio-it.cn/#/FAQ.

## Usage of Cancer Antigen Platform

A chosen peptide could be explored in this comprehensive platform. Specific processes can refer to Supplementary Figure S3. We also provide multiple useful tools, such as the sequence alignment tool, which provides the opportunity to explore and compare new cancer antigens with prior knowledge in our database. In addition, we also facilitated online MHC–peptide structure modeling, exploring the peptide–MHC binding and/or pMHC–TCR structure. For specific usage cases, refer to the description in Supplementary Figure S4. It can also be locally run by users with an algorithmic foundation. Users can refer to the source code at https://github.com/yujijun/pMHC_TCR_binding.

## Discussion

Given the problems in the prediction of cancer antigens, especially neoantigens, we systematically organized and explained the usage of datasets. All these datasets and their usage proposals will greatly promote the accuracy of tumor neoantigen prediction, especially in machine learning or deep learning algorithm scenarios. However, in the process of algorithm development, developers must pay attention to the characteristics of each dataset. For example, pMHC MS datasets lack negative observations (peptides that do not bind), posing challenges in creating predictive models (Villani et al., 2018). In the situation of immunogenicity study of antigens, the T-cell epitope datasets may be better than peptide–MHC binding or MS datasets because not all peptides presented by MHC can provoke T-cell activation. Multiple datasets and strategies can be integrated to improve overall results. For example, combining pMHC MS datasets into MHC-binding datasets might make prediction of peptide–MHC binding more accurate.

Except for considering the attributes of datasets, many methods of improving performance inherent in machine learning or deep learning can also be considered. De-redundancy of datasets may improve the scalability of the algorithm and also make a more accurate evaluation. In some of the datasets mentioned before, negative will be much larger than positive. Many down-sampling methods could be used to prevent model prediction bias; in the case of insufficient training data, users can generate simulated datasets as previously mentioned.

In addition to the detailed introduction of the data and algorithms mentioned previously, a one-stop interaction platform was established, which is convenient for all cancer antigen researchers to conduct online exploration of cancer antigen properties, such as the affinity and hydrophobicity, pMHC or PMHC–TCR docking characteristics, and key binding sites. This is very important for the re-excavation of existing cancer antigen information. Information is still not detail enough for readers to explore at a more specific. Therefore, we are looking forward to the blueprint mentioned in the CEDAR database (Koşaloğlu-Yalçın et al., 2021). CEDAR was built based on IEDB (Vita et al., 2019), including all cancer-specific epitope data from various T-cell and B-cell experiments, MHC-binding assays, and MHC ligandomics by MS. Simultaneously, the peptide information will be associated with biologically, immunologically, and clinically relevant information, and a fine-tuned classification and retrieval mechanism will be established. For researchers without programming experience, the information on relevant epitope terms can be accurately investigated online, which facilitates experiments based on prior knowledge. At the same time, it provides online calculation and objective evaluation between different epitope tools, which greatly reduces the difficulty of selecting epitope prediction tools for those who have no programming experience and are unfamiliar with algorithms.

In this study, we curated the most comprehensive datasets of verified cancer antigens and systematically explained the usage of the various datasets in neoantigen algorithm development and then established the online exploration platform for cancer antigens and integrated useful tools to conveniently and comprehensively investigate. We believe all these efforts will support researchers in cancer antigens with or without programming experience. We will continue to improve our platform to make it more informative and convenient to use.

## Data Availability

The datasets for this study can be found at http://cad.bio-it.cn/#/Download, further inquiries can be directed to the corresponding authors.
