# Supplementary material for: CAD v1.0: Cancer Antigens Database Platform for Cancer Antigen Algorithm Development and Information Exploration
Source: Front Bioeng Biotechnol. 2022 May 12;10:819583. doi: 10.3389/fbioe.2022.819583 (PMC9133807; doi:10.3389/fbioe.2022.819583)
Supplement: Supplementary file 1 [file Table1.docx]

Supplementary Table 1 | Benchmark datasets for neoantigen pipeline training and validation

| ID | Reference | Title | Journal | Data project | Download link |
| --- | --- | --- | --- | --- | --- |
| 1 | 26516200 | Immunogenicity of somatic mutations in human gastrointestinal cancers | Science, 2015 | PRJNA298330 | https://www.ncbi.nlm.nih.gov/bioproject/?term=PRJNA298330 |
| 2 | 25837513 | A dendritic cell vaccine increases the breadth and diversity of melanoma neoantigen-specific T cells | Science, 2015 | PRJNA278450 | https://www.ncbi.nlm.nih.gov/bioproject/?term=PRJNA278450 |
| 3 | 26901407 | Prospective identification of neoantigen-specific lymphocytes in the peripheral blood of melanoma patients | nature medicine, 2015 | PRJNA298310 | https://www.ncbi.nlm.nih.gov/bioproject/?term=PRJNA298310 |
| 4 | 26389673 | Isolation of neoantigen-specific T cells from tumor and peripheral lymphocytes | The Journal of Clinical Investigation, 2015 | SRP062169 | https://trace.ddbj.nig.ac.jp/DRASearch/study?acc=SRP062169 |
| 5 | 28423700 | HLA class I loss in metachronous metastases prevents continuous T cell recognition of mutated neoantigens in a human melanoma model | Oncotarget, 2017 | SRP068803 | https://trace.ddbj.nig.ac.jp/DRASearch/run?acc=SRR3136204 |
| 6 | 32087727 | neoANT-HILL: an integrated tool for identification of potential neoantigens | BMC Med Genomics, 2020 | E-GEUV-1 | https://www.ebi.ac.uk/arrayexpress/experiments/E-GEUV-1/ |
| 7 | 26997480 | Genomic and Transcriptomic Features of Response to Anti-PD-1 Therapy in Metastatic Melanoma | Cell, 2016 | PRJNA312948 | https://www.ncbi.nlm.nih.gov/bioproject/?term=PRJNA312948 |
|  |  |  |  | PRJNA307199 | https://www.ncbi.nlm.nih.gov/bioproject/PRJNA307199 |
|  |  |  |  | PRJNA343789 | https://www.ncbi.nlm.nih.gov/bioproject/PRJNA343789 |
| 8 | 27667683 | Loss of IFN-γ pathway genes in tumor cells as a mechanism of resistance to anti-CTLA-4 therapy | Cell, 2016 | PRJNA357321 | https://www.ncbi.nlm.nih.gov/bioproject/PRJNA357321 |
| 9 | PMC4993154 | Mutational landscape determines sensitivity to PD-1 blockade in non-small cell lung cancer | Science, 2015 | PRJNA293912 | https://www.ncbi.nlm.nih.gov/bioproject/?term=PRJNA293912 |
| 10 | 28251903 | Integrated molecular analysis of tumor biopsies on sequential CTLA-4 and PD-1 blockade reveals markers of response and resistance | Sci Transl Med, 2017 | PRJNA369259 | https://www.ncbi.nlm.nih.gov/bioproject/?term=PRJNA369259 |
| 11 | 26317466 | Inhibiting DNA Methylation Causes an Interferon Response in Cancer via dsRNA Including Endogenous Retroviruses | Cell, 2015 | PRJNA305077 | https://www.ncbi.nlm.nih.gov/bioproject/?term=PRJNA305077 |
| 12 | 25409260 | Genetic basis for clinical response to CTLA-4 blockade in melanoma | N Engl J Med, 2014 | PRJNA306070 | https://www.ncbi.nlm.nih.gov/bioproject/?term=PRJNA306070 |
| 13 | 26359337 | Genomic correlates of response to CTLA-4 blockade in metastatic melanoma | Science, 2015 | PRJNA82745 | <https://www.ncbi.nlm.nih.gov/bioproject/?term=PRJNA82745> |
| 14 | 27433843 | Mutations Associated with Acquired Resistance to PD-1 Blockade in Melanoma | N Engl J Med, 2016 | PRJNA324705 | https://www.ncbi.nlm.nih.gov/bioproject/?term=PRJNA324705 |
| 15 | 23629695 | Discovery and mass spectrometric analysis of novel splice-junction peptides using RNA-Seq | Mol Cell Proteomics, 2013 | GSE45428 | https://www.ncbi.nlm.nih.gov/geo/query/acc.cgi?acc=GSE45428 |
| 16 | [27869121](https://www.ncbi.nlm.nih.gov/pubmed/27869121) | Direct identification of clinically relevant neoepitopes presented on native human melanoma tissue | Nature Communication, 2016 | EGAS00001002050 | <https://www.omicsdi.org/dataset/ega/EGAS00001002050> |
