# Supplementary material for: CAD v1.0: Cancer Antigens Database Platform for Cancer Antigen Algorithm Development and Information Exploration
Source: Front Bioeng Biotechnol. 2022 May 12;10:819583. doi: 10.3389/fbioe.2022.819583 (PMC9133807; doi:10.3389/fbioe.2022.819583)
Supplement: Supplementary file 3 [file Image2.PDF]

Supplementary Figure 2

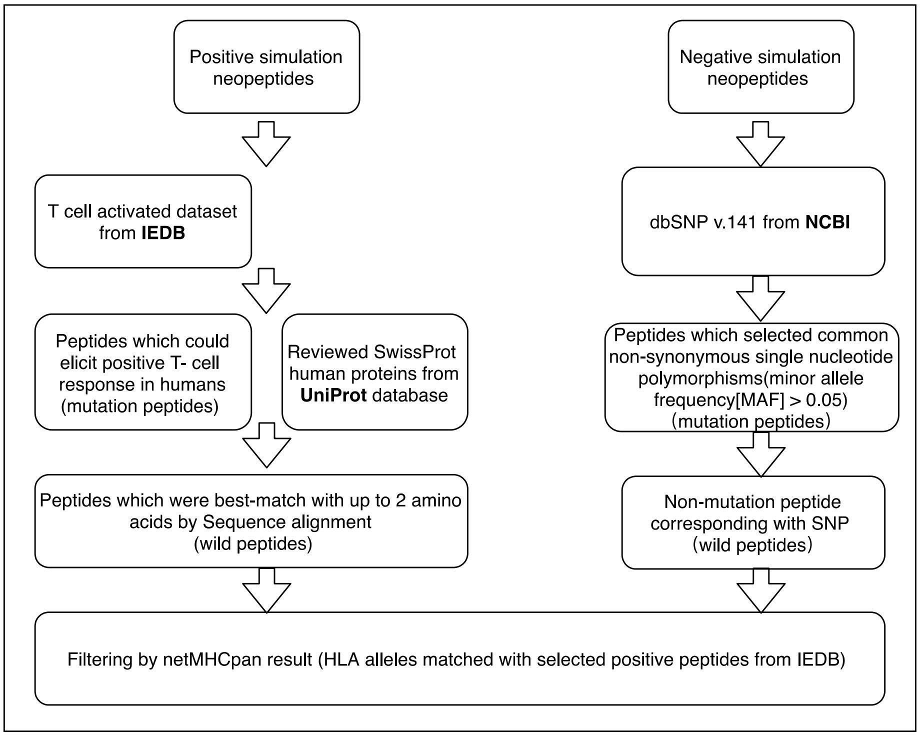

SUPPLEMENTARY FIGURE 2 | Schematic diagram of simulation neoantigen generation process, detail description could be found in github (https://github.com/yujijun/NeoSimData)
