# Supplementary material for: CAD v1.0: Cancer Antigens Database Platform for Cancer Antigen Algorithm Development and Information Exploration
Source: Front Bioeng Biotechnol. 2022 May 12;10:819583. doi: 10.3389/fbioe.2022.819583 (PMC9133807; doi:10.3389/fbioe.2022.819583)
Supplement: Supplementary file 4 [file Image3.PDF]

## Supplementary Figure 3

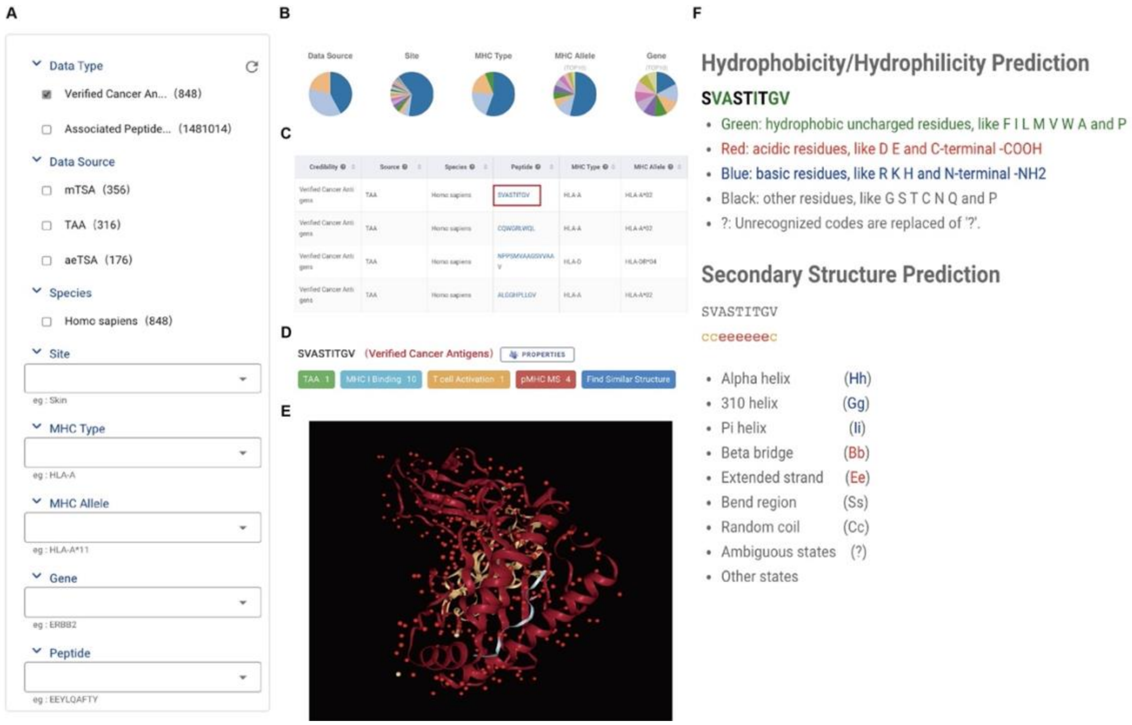


**SUPPLEMENTARY FIGURE 3 |** Usage case of exploration of cancer antigen properties; We can click “Verified Cancer Antigen” on the left border in SEARCH page to select cancer antigens (A), and then we can see various types of information statistics about Site, and top10 MHC allele and gene in top of this page (B). The main table shows the detailed information of peptides (C), there are many links that can be jumped for more information explanation, such as clicking on a peptide itself, we can see all the information related to with this peptide (D), the “SVASTITGV” peptide has been verified by different experiments in different literatures. It is worth noting that we provide MHC-peptide or MHC-peptide-TCR structure collected from PDB if identity or similarity peptides were existed (E), users can directly explore three-dimensional structure of those compounds on the website (E) or use the structure as a template and use the one provided by this website (In TOOLS page) or other homologous modeling tools for structure modeling. It is even possible to explore the structure of the site before and after the mutation, which is of great significance for the study of mutations for the binding of new antigens. Furthermore, the hydrophilicity and hydrophobicity of the peptide and the secondary structure information were also taken into account in our dataset, click the properties button, this information will be displayed in a pop-up box (F)
