# Supplementary material for: CAD v1.0: Cancer Antigens Database Platform for Cancer Antigen Algorithm Development and Information Exploration
Source: Front Bioeng Biotechnol. 2022 May 12;10:819583. doi: 10.3389/fbioe.2022.819583 (PMC9133807; doi:10.3389/fbioe.2022.819583)
Supplement: Supplementary file 5 [file Table2.docx]

Supplementary Table 2 Data statistics and usage description of the datasets

|  | **Data Name** | **Number** | **Description** | **Usage** | **Usage Case** |
| --- | --- | --- | --- | --- | --- |
| Cancer Antigens | mTSAs (Neoantigens) | 267 | Mutated tumor-specific antigens, only exist in tumor cells, which are tumor-specific antigens and hardly shared by different patients. | These datasets could be used to explore all shared or specific antigens in certain cancer and be suitable for potential cancer antigens validation | Bao *et al* (Bao et al., 2021) Valid the Potential Neoantigens by Public Data, just like IEDB and TSNAdb, CTDatabase. Our database can also serve as such a database to verify the results of cancer antigens predictions |
|  | aeTSAs | 229 | Aberrantly expressed tumor-specific antigens, that are not expressed in adult somatic cells, also specific antigens and might be shared by different patients. |  |  |
|  | TAAs | 325 | Tumor-associated antigens, proteins that exist in normal tissues but also overexpressed in cancers, which are not tumor-specific antigens, might be shared among patients. |  |  |
|  | Simulated Neopeptides | 6787 (only HLA A*0201) | Simulated datasets are an updated version of construction of neopeptides datasets mentioned by Kim *et al* (Kim et al., 2018a) | This dataset could be used training neoantigen immunogenicity prediction modeling | Kim *et al* (Kim et al., 2018a) had used this kind of dataset to make the Determination of immunogenic neo-antigens based on machine learning methods. |
| MHC Binding | MHC I Binding | 452648 | Validated MHC class I or MHC class II and peptides binding datasets were from IEDB, which have been cleaned and tidied up. In the meantime, human organism peptides were filtered and stored in our database. | These datasets are suitable for algorithms development of binding affinity and algorithms improvement based on similarity | Just like many tools linked in IEDB (Dhanda et al., 2019; Vita et al., 2019); Furthermore, Balachandran *et al* (Balachandran et al., 2017) and Łuksza *et al* (Luksza et al., 2017) developed a new approach for assessing whether a tumor is immunogenically based on sequence similarity between the predicted neoantigens and a database of immunogenic epitopes |
|  | MHC II Binding | 117305 |  |  |  |
| T cell activation | T cell Epitopes | 66151 | The experimentally verified T cell epitope datasets from IEDB and incomplete terms have been removed. In the meantime, human organism peptides were filtered and stored in our database. | These datasets could be used for algorithms development of T cell epitopes prediction algorithms |  |
| Antigens | Antigens with TCRseq info | 60267 | peptides of antigens with TCR sequences information from the VDJdb database (Bagaev et al., 2020) | These datasets could be used for Predicting recognition between T cell receptors and epitopes; Exploring binding properties between antigens and TCR. | Jokinen *et al* (Jokinen et al., 2019) developed the TCRGP algorithm for Predicting recognition between T cell receptors and epitopes based on these kinds of datasets |
| MS | pMHC MS | 509536 | pMHC MS datasets were from SysteMHC. the MS data containing information on MHC-peptide interactions. | These datasets (Creech et al., 2018) could be used to improve the prediction accuracy of MHC and peptides binding | NetMHCpan-4.1 and NetMHCIIpan-4.0 (Reynisson et al., 2021) improve predictions of MHC antigen presentation by integration of MS MHC eluted ligand data |
| Benchmark datasets | Benchmark | 16 | Published public datasets include the entire process from the original sequence dataset, predicted neoantigens, to experimentally verified immunogenic peptides | Benchmark datasets could be used for testing and verification of neoantigen pipelines. | pTuneos (Zhou et al., 2019a) and neoepiscope (Wood et al., 2020) evaluated the performance of the pipelines by published melanoma patients; PVACtools (Hundal et al., 2020) also reanalyzed raw sequencing data from published studies that had performed immunologic validation of candidate neoantigens; |
