# Supplementary material for: CAD v1.0: Cancer Antigens Database Platform for Cancer Antigen Algorithm Development and Information Exploration
Source: Front Bioeng Biotechnol. 2022 May 12;10:819583. doi: 10.3389/fbioe.2022.819583 (PMC9133807; doi:10.3389/fbioe.2022.819583)
Supplement: Supplementary file 6 [file Table3.docx]

Supplementary Table 3 | Datasets or tools mentioned in our dataset

| **Type** | **Function** | **Name** | **Link** |
| --- | --- | --- | --- |
| Database | Data Resource | IEDB | https://www.iedb.org/ |
| Database | Data Resource | dbSNP | https://www.ncbi.nlm.nih.gov/snp/ |
| Database | Data Resource | SysteMHC | <https://systemhcatlas.org> |
| Database | Data Resource | Uniprot | https://www.uniprot.org/uniprot/?query=reviewed:yes |
| PDB | Data Resource | PDB | https://www.rcsb.org/ |
| Tools | Peptide Properties | PEPTIDE 2.0 | <https://www.peptide2.com/N_peptide_hydrophobicity_hydrophilicity.php> |
| Tools | Peptide Properties | GOR IV SECONDARY STRUCTURE PREDICTION METHOD | https://npsa-prabi.ibcp.fr/cgi-bin/npsa_automat.pl?page=/NPSA/npsa_gor4.html |
| Tools | Structure Modelling | Modeller | https://salilab.org/modeller/ |
| Tools | MHC Class I Processing | MHC-I Processing Predictions | http://tools.iedb.org/processing/ |
| Tools | MHC Peptide Binding Affinity Prediction | MHC-I Binding Prediction | http://tools.iedb.org/mhci/ |
| Tools | MHC Peptide binding affinity prediction | MHC-II Binding Prediction | http://tools.iedb.org/mhcii/ |
| Tools | Neoantigen Prediction Pipeline | [NeoPredPipe](https://github.com/MathOnco/NeoPredPipe) | https://github.com/MathOnco/NeoPredPipe |
| Tools | Neoantigen Prediction Pipeline | [NeoantigenR](https://github.com/ICBI/neoantigeR) | https://github.com/ICBI/neoantigeR |
| Tools | Neoantigen Prediction Pipeline | [CloudNeo](https://github.com/TheJacksonLaboratory/CloudNeo) | https://github.com/TheJacksonLaboratory/CloudNeo |
| Tools | Neoantigen Prediction Pipeline | [Neoantigen-vaccine-pipeline](https://github.com/openvax/neoantigen-vaccine-pipeline) | https://github.com/openvax/neoantigen-vaccine-pipeline |
| Tools | Neoantigen Prediction Pipeline | [PVACTools](https://pvactools.readthedocs.io/en/latest/pvacseq.html) | https://pvactools.readthedocs.io/en/latest/pvacseq.html |
