# Supplementary material for: CAD v1.0: Cancer Antigens Database Platform for Cancer Antigen Algorithm Development and Information Exploration
Source: Front Bioeng Biotechnol. 2022 May 12;10:819583. doi: 10.3389/fbioe.2022.819583 (PMC9133807; doi:10.3389/fbioe.2022.819583)
Supplement: Supplementary file 7 [file Image1.PDF]

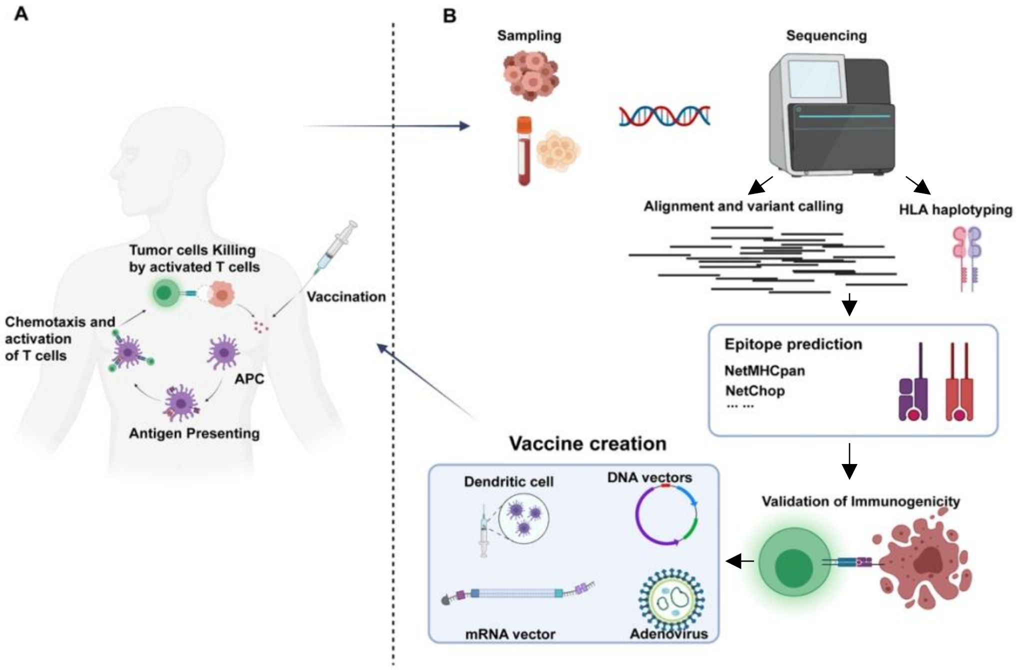

SUPPLEMENTARY FIGURE 1 | Schematic diagram of neoantigen prediction pipeline and tumor killing process. (A) The process by which a vaccine enters the body and performs its tumor-killing function; (B) neoantigen prediction pipeline.
