# Supplementary material for: CAD v1.0: Cancer Antigens Database Platform for Cancer Antigen Algorithm Development and Information Exploration
Source: Front Bioeng Biotechnol. 2022 May 12;10:819583. doi: 10.3389/fbioe.2022.819583 (PMC9133807; doi:10.3389/fbioe.2022.819583)
Supplement: Supplementary file 2 [file Image4.PDF]

# Supplementary Figure 4

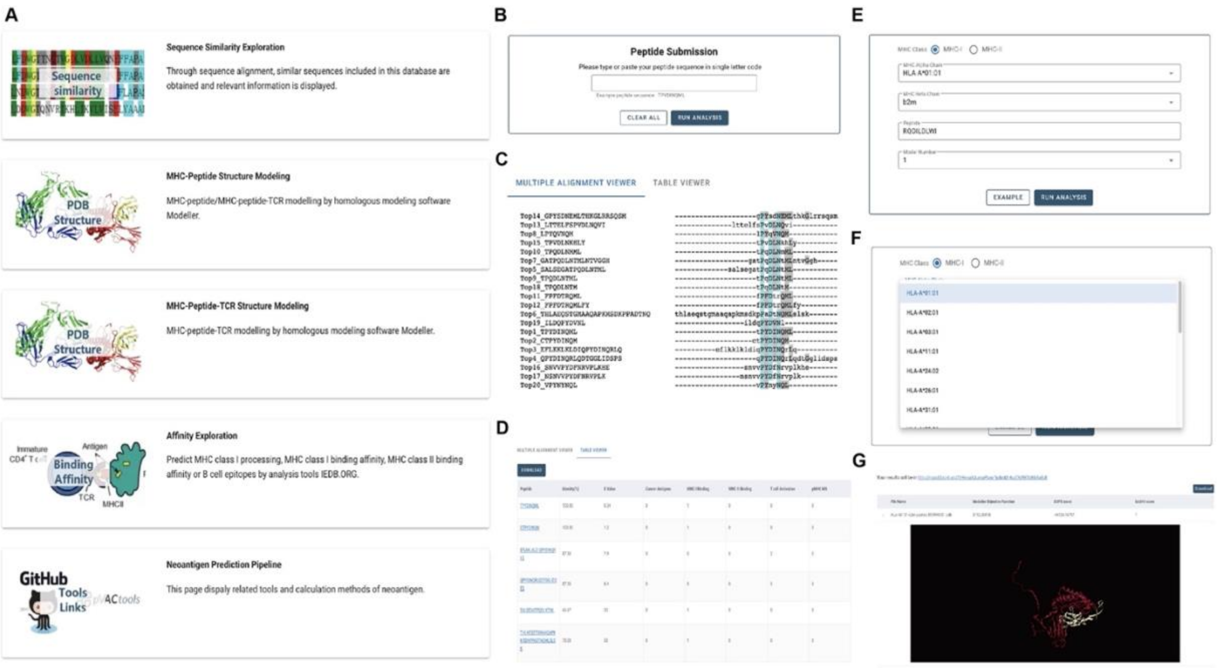


**SUPPLEMENTARY FIGURE 4 |** Usage case of Tools; There were five types of tools on this website that were integrated or connected to the website (A). The first one is the sequence alignment software. Users can enter an interested peptide in the website or any peptides they want to explore and compare with peptides in the database (B). The alignment information was exampled in C and D, this is of great significance for connecting the peptides outside the library with inside. Click the peptides information in C to jump directly to the detailed information of the specific peptide to explore the peptide information in detail. In addition to the sequence level, we can also explore peptides at the structure level by tools of “MHC-Peptide Structure Modeling” or “MHC-Peptide-TCR Structure Modeling”. For example, click to enter the “MHC-Peptide Structure Modeling” interface, users could select an HLA allele from 30 HLAs (top 10 of most common HLA-A, HLA-B, and HLA-C alleles), and enter a peptide to be predicted, submit and jump to the result interface (G). The operation may take a few minutes. If users want to close the interface, please remember to record the web page link, which can be used to browse result online or download to watch. At the same time, it also supports locally running by users with algorithmic foundation, if so, users could consult the author for source code (https://github.com/yujijun/pMHC_TCR_binding).
